# Impact of repeated preheating of bulk-fill resin composite on postoperative hypersensitivity; a randomized controlled clinical trial

**DOI:** 10.1186/s12903-024-04170-4

**Published:** 2024-04-15

**Authors:** Mahmoud Elkady, Safaa Helmy Abdelhakim, Mona Riad

**Affiliations:** 1 Conservative Dentistry Department, Faculty of Dentistry, Assiut University, Assiut, Egypt; 2 Conservative Dentistry Department, Faculty of Dentistry, Minia University, Minia, Egypt; 3 Conservative Dentistry Department, Faculty of Dentistry, Cairo University, Cairo, Egypt

**Keywords:** Bulk-fill resin composite, Repeated preheating, Postoperative hypersensitivity, Visual analogue scale

## Abstract

**Background:**

This clinical study was conducted aiming to evaluate the impact of repeated preheating of bulk-fill resin composite on postoperative hypersensitivity.

**Methods:**

A total of 105 eligible, consenting adults were recruited. Patients had posterior teeth suffering from proximal decay with no signs of irreversible pulpitis. Patients were prepared for Class II restorations and restored with bulk-fill resin composite. Patients were randomized into three groups of 35 patients according to the number of preheating cycles for the resin composite syringe used; **group I**: no preheating; control group at room temperature, **group II**: Resin composite preheated once, and **group III**: Resin composite preheated ten cycles. Patients were assessed for postoperative dentin hypersensitivity using the visual analogue scale (VAS) at three-time intervals: day one, one week and by the end of one month after restorative treatment. Statistical analysis was performed; ANOVA with a single factor was used to test for significance at a p value ≤ 0.05. For nonparametric data, the Kruskal‒Wallis test was used to compare the three testing groups. Friedman’s test was used to study the changes within each group. Dunn’s test was used for pairwise comparisons when the Kruskal‒Wallis test or Friedman’s test was significant.

**Results:**

The scores of the three groups through the three time intervals were almost zero except for the first day where VAS scores were recorded with maximum score of 3 for groups I and II. Groups II and III; there was no statistically significant change in hypersensitivity scores by time with *P*-values 0.135 and 0.368, respectively. However, for group I there was a significant difference from VAS score recorded on first day and the two following time intervals.

**Conclusion:**

The repeated preheating cycles of bulk-fill resin composite prior to curing had no adverse effect on the patients regarding postoperative dentin hypersensitivity. This information could be of utmost significance, as the same resin composite syringe can undergo numerous preheating cycles clinically before it is completely consumed with the advantage of improvement on the handling properties.

**Trial registration:**

The protocol of the current study was registered at www.clinicaltrials.gov, with the identification number NCT05289479 on 21/03/2022. All procedures involving human participants were performed in accordance with the ethical standards of the Research Ethics Committee of the Faculty of Dentistry, Minia University, Egypt, under the approval number 73/440 on 11/09/2020.

## Background

The modern concepts of adhesive dentistry to both enamel and dentin made using dental resin composite (RC) are one of the ruling restorative materials nominated for both aesthetic and restorative purposes either in anterior or posterior teeth. Servicing a RC restoration that can fulfil the criteria of success in both aesthetics and functionality is the target for all dental professionals. However, the dissatisfaction of patients due to pain after regular restorative treatment has always been a concern. Many factors can provoke this pain, including gingival inflammation, periodontitis, or postoperative dentin hypersensitivity, and the latter could be considered the main cause of dissatisfaction [1–3].

Dental professionals are constantly looking for dental restoration procedures that do not cause postoperative dentin hypersensitivity [4]. Unfortunately, many possibilities have been reported, such as adhesive protocols, contamination during the restorative procedure, shrinkage stresses or marginal leakage [5–7]. The prolonged complaints of postoperative dentin hypersensitivity caused by marginal leakage have led to poor outcomes in the assessment of dental restorations, which in turn push the dental profession for undesired dental restoration remakes [3, 8, 9].

Dental RCs suffer from polymerization shrinkage of approximately 1–6% by volume [10]. This volumetric shrinkage is translated to massive stresses on the hybrid layer, which could be destroyed due to these stresses [11]. Many techniques are performed in the dental profession to diminish this kind of stress. Incremental packing of RC is one of the major steps performed to compensate for both polymerization shrinkage of the RC material and the resultant polymerization shrinkage stresses; however, it is believed that it is a time-consuming procedure and highly dependent on the dental operator’s skills [12, 13]. Bulk-fill RCs are also considered to compensate for both shrinkage stresses and time consumption [14–16]. Nowadays, bulk-fill RC is thought to be an excellent substitute for traditional RC, offering clinical results that are equivalent for up to ten years [17, 18]. Furthermore, patients’ postoperative discomfort would not be affected adversely by using bulk-fill RC for restoring dental cavities [19, 20]. On the other hand, some literature had found that bulk-fill technique has many concerns regarding the degree of conversion, aesthetics, mechanical properties and even polymerization shrinkage [21, 22]. In addition, the less satisfying handling properties encountered by operators compared to conventional RC.

Dentists have been requested over the years to chill the RC under 8 °C, until just before use, as well as in between patients. The debate over whether to refrigerate or preheat RC before use continues [23]. One of the obvious challenges that is encountered by dental professionals during RC application is the adaptation capabilities of the material, which differ from one product to another. The ability of dental professionals to drag the RC paste towards the walls and floor of the prepared cavities with a pliable but low-viscosity material was a concern that had been addressed by many manufacturers [24]. However, some of the RC pastes are difficult to manipulate and modify. Recently, some manufacturers recommended preheating of RC before use to overcome this drawback “Filtek One-Bulk, 3m Deutschland, Germany, and VisCalor Bulk, Voco, Cuxhaven, Germany”.

Modifying the rheological properties of bulk-fill RCs by heating before application could facilitate their application. Preheating before placement may have significant clinical benefits, such as reduced film thickness, enhanced adaptation and decreased microleakage, shorter curing times, increased hardness, and sufficient flowability, which in turn secures superior manipulation and adaptation to the prepared cavity walls [25–27]. Elevating the temperature of the RC from 50 to 70 °C before application can also aid in increasing the degree of conversion of the dental RC through the free movement of the monomers, allowing them to meet each other and form longer polymeric chains, in addition to enabling them to achieve better cross-linking between polymeric chains [28]. Despite these enhanced features, RC preheating is not a regularly used procedure. The lack of adequate clinical evidence supporting the use of warmed RCs among dental professionals is one potential explanation for their hesitation. Laboratory investigations to gauge a restorative material’s effectiveness before clinical studies are more crucial, as a number of factors, including mastication forces, temperature changes, humidity changes, and salivary enzymes, could affect how well a restorative material functions overall [29]. Laboratory studies on preheated RCs have demonstrated that these composites have improved properties. However, their improved rheological properties may or may not reduce postoperative sensitivities. There has only ever been one documented randomized controlled clinical trial of postoperative dentin hypersensitivity to assess the therapeutic efficacy of warmed RCs, but for single preheating cycle [30].

For economic purposes, RC syringes rather than capsules have always been chosen by dental professionals. Under clinical conditions, a syringe containing RC undergoes several thermal cycles as it is repeatedly used for the restoration of several cavities. The goal of the current study, which evaluated the impact of preheating RC on the clinical performance of class II restorations over the course of a month, was to provide more evidence in this research’s viewpoint and whether repeated preheating offers additional benefits in clinical settings. The null hypothesis tested was that there is no difference in postoperative hypersensitivity between bulk fill RC preheated to 68ºC and subjected to one or ten preheating cycles at placement and room temperature RC.

## Methods

### Study design

The description of the experimental design followed the Consolidated Standards of Reporting Trials statement [31]. The present study was a double-blind (patients and examiner) randomized clinical trial anticipating a single-mouth design. Three parallel groups with a 1:1:1 allocation ratio was determined by using online software (www.sealedenvelope.com). The selected carious teeth were sporadically divided into three groups as each patient received a single RC restoration; Group I: RC at room temperature, Group II: the RC syringe underwent one preheating cycle before use, and Group III: the RC syringe underwent ten preheating cycles. Thirty-five teeth were included in each group.

### Sample size calculation

This power analysis used marginal integrity after 12 months as the primary outcome. Based upon the results of Kurdi R and Abboud SA (2016) [32], the bulk-fill composite had 13 cases with an α score and 4 cases with a β score. The effect size (w) was 0.53. Using an α level of (5%) and β level of (20%), i.e., power = 80%; the minimum estimated sample size was 28 cases. The sample size was increased to 35 cases for each group to compensate for a drop-out rate of 25%. Sample size calculation was performed using G*Power Version 3.1.9.2 [33].

### Patient selection

From patients seeking dental treatment in the Conservative Department Clinic at the Faculty of Dentistry, Minia University, only 105 patients meeting the inclusion criteria were included in the current study. These patients were either dental students or employees in the department. Included patients were between 18 and 40 years of age and had carious lesions in both proximal and occlusal surfaces that were detected clinically and evaluated by X-ray to note the extension of the carious lesions. only extension of carious lesion, not exceeding half the thickness of dentin “D1 and D2”, was included. The patients were to have good oral hygiene and show no spontaneous pain or orofacial pain. The selected tooth needed to give a positive response to testing with an electric pulp tester, have normal and full occlusion, and have opposing natural teeth with no restorations. The selected teeth had healthy gingival tissues with no recession or alveolar bone loss.

However, patients who had deeper cavities with more than half the thickness of dentin toward the pulp “D3 and D4”, heavy bruxism habits, engaged in clenching, showed evidence of wear facets on teeth, taking analgesics that could alter their normal pain perception level, suffering occlusal disturbances, experiencing temporo-mandibular joint problems, or undergoing orthodontic treatment were excluded before any procedure was performed. Only patients who were meeting the inclusion criteria were included in the study. Initially, illustration of the procedure was performed on each patient included in the current study. Each patient signed a consent form before participation.

### Randomization and blinding

Simple randomization was performed for the prepared cavities to determine which tooth received which intervention. A box containing three different colored cards “yellow, blue and green” with a total of 105 cards was selected randomly by a blinded assisting operator not involved in the study who prepared the envelopes and gave each patient a random numbered and colored card. The main operator was not blinded to the material assignment. However, the patients always remained blind to the allocation. All patients were listed with the given randomized numbers; those with numbers from 1 to 35 with the yellow cards were allocated in group I, those with numbers from 36 to 70 with the blue cards were allocated in group II, and those with numbers from 71 to 105 with the green cards were in group III.

### Clinical procedure

Initially, illustration of the procedure was performed on each patient included in the current study. Each patient was anaesthetized using the local anaesthetic solution Artinibsa 4% articaine with 1/100,000 epinephrine. Rubber dam isolation was applied with multiple isolation techniques, and cavity preparation was performed using a 1:5 steady torque hand piece (NSK, Japan) with copious water cooling followed by excavation of remaining caries using tungsten carbide burs (Komet, Brasseler GmbH Co. KG) at a low speed and sharp excavators (mailfaire 57/58, Switzerland). The neighbouring tooth was guarded using a wedge guard (Palodent, Dentsply Sirona, Germany) using 330, 245 and 329 burs and finished with yellow coded stones (Komet, Germany).

After shade selection, matrixing was performed using a suitable sectional matrix and wooden wedges in addition to a separating ring (NiTinol rings, re-Invent, USA). A selective etching technique was utilized in the present study; first enamel etching using 35% phosphoric acid (HV Bisco, USA) was performed for 15 s, followed by rinsing with water for 20 s and drying with air free of moisture and oil for 5 s. Then, adhesive (Scotchbond Universal Plus, 3 M Deutschland, Germany) was applied in a single drop with active application by scrubbing action for 20 s on both enamel and dentin and air thinned using oil-free air flow for 5 s according to the manufacturer’s instructions. A fully charged light-curing device (Radii Plus, SDI, Australia) with an output intensity of 1500 mW/cm^2^ was applied for 20 s. The intensity of the light-curing unit was measured periodically using the integrated radiometer of the same light-curing device to ensure the light intensity.

Room temperature Bulk-fill RC (Filtek One Bulk, 3 M Deutschland, Germany) was packed to the prepared Class II cavities of patients in the control group. In the groups II and III, a HeatSync RC warming kit (Bioclear, USA) was used in accordance with the manufacturer’s instructions to heat RCs. The RC heater (Bioclear HeatSync) was turned on for at least 15 min before insertion of the RC syringes that were to be heated. The syringes were left in the syringe port for at least 10 min to reach a temperature of 68ºC each time the RC was preheated. It took 15 min to reach and stabilize the selected preset temperature (68ºC). The entire body of the syringe was inserted completely in its port for each preheating cycle.

For group II, the RC syringe was warmed to 68ºC one time only just before use; however, in group III, the RC syringe was warmed to 68ºC and cooled to room temperature for 9 successive cycles, to be ready for a tenth time of preheating just before use. The teeth were restored incrementally up to two increments, with a maximum of 2 mm thickness each. The RC syringe was removed from the heating device, and gold-plated instruments were used to apply the RC immediately in no more than 10 s. Consequently, each increment was light cured for 10 s. The operator was strictly applying and curing each RC increment in only 20 s to control heat loss. After handling each increment, the RC syringe was inserted immediately back again in the heating device. After finishing the restorative procedure, rubber dam isolation was removed, all the restorations were checked for any premature contact, and were finished and polished using EVE DIACOMP Plus OccluFlex-impregnated rubber cups and impregnated brushes (Optishine, Kerr Switzerland).

### Hypersensitivity testing

**Fig. 1:**
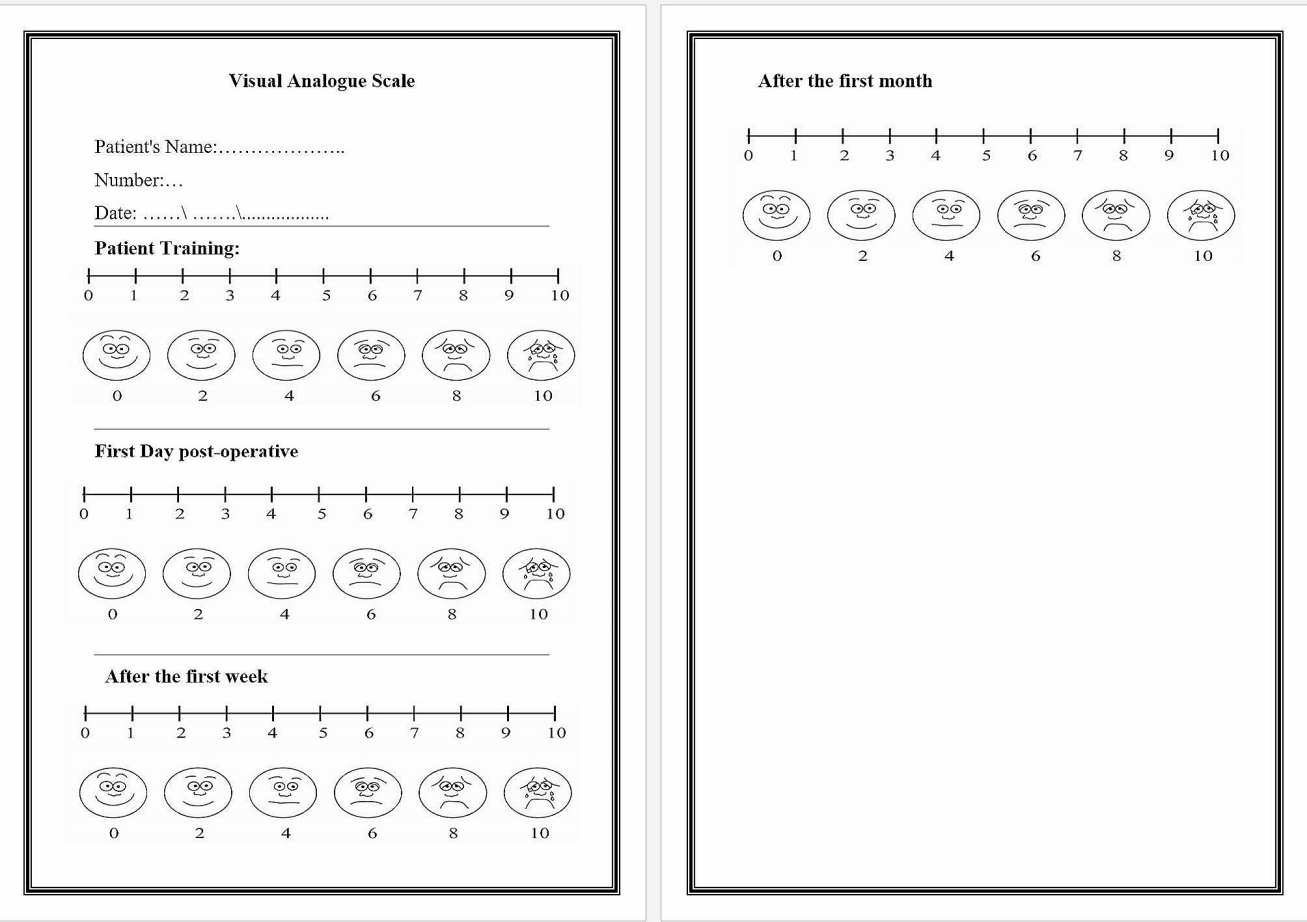
VAS chart handled by the enrolled patients

A Visual Analog Scale (VAS) for the evaluation of postoperative hypersensitivity was used. All patients received a VAS chart (Fig. 1) and were trained to mark it on day one, after one week and by the end of one month after the restorative treatment. The patient returned the VAS test chart every follow-up period in a closed envelope with the patient number written on the envelope so that the data could be entered an Excel sheet (Microsoft Office 365) by a trained blinded operator. In case of the presence of moderate or severe postoperative pain, the patients were instructed to take analgesic tablets, asked to record the incidence of intake and the number of analgesic tablets taken, and then return to the clinic as soon as possible. The clinical intraoral photographs were taken at all recall periods.

### Statistical analysis

Numerical data were explored for normality by checking the distribution of data and using tests of normality (Kolmogorov‒Smirnov and Shapiro‒Wilk tests). Age data showed a normal (parametric) distribution, while hypersensitivity (VAS) scores showed a nonnormal (nonparametric) distribution. Data are presented as median, range, mean and standard deviation (SD) values. For parametric data, one-way ANOVA was used to compare mean age values among the three groups. For nonparametric data, the Kruskal‒Wallis test was used to compare the three groups. Friedman’s test was used to study the changes within each group. Dunn’s test was used for pairwise comparisons when the Kruskal‒Wallis test or Friedman’s test was significant. The significance level was set at *P* ≤ 0.05. Statistical analysis was performed with IBM SPSS Statistics for Windows, Version 23.0. Armonk, NY: IBM Corp.

## Results

All the included patients did not record any problem in handling or sending the VAS scores throughout the follow-up period. If any obstacle hindered the physical attendance of any included patient to handle their VAS chart, the patient was asked to send it through any means of electronic messaging. Demographic data analysis showed no statistical significance related to any aspect related to age, gender, position or whether the tooth restored was premolar or molar (Table 1). As an overview, all included patients did not complaint from severe pain demanding any interpretation. Out of score 10, the highest score recorded by patients in all groups was only 3 for groups I and II, while group II did have highest score of 2 on the first day. Over the whole follow up period, after placement of the RC restorations, data collection and statistical analysis, there was no statistically significant difference in the VAS scores between the three different groups when compared with each other at different recall times (1st day, 1st week and 1st month) (Table 2).

Regarding Group I, there was a statistically significant change in post-operative hypersensitivity scores over time (*P* value = 0.024, effect size = 0.265). Pairwise comparisons between time periods revealed that there was a statistically significant decrease in hypersensitivity scores after one week followed by a non-statistically significant change from one week to one month. Moreover, for Groups II and III, there was no statistically significant change in hypersensitivity scores over time (*P* value = 0.135, effect size = 0.143 and *P* value = 0.368, effect size = 0.071, respectively) (Table 3).

**Table 1:** Mean, standard deviation (SD), and results of one-way ANOVA test and Chi-square test for comparison between demographic data in the three groups

	Group I (*n* = 35)	Group II (*n* = 35)	Group III (*n* = 35)	P value
**Age (years)**				0.329
Mean (SD)	30.3 (6.4)	29.7 (5.3)	31.9 (7.2)	
**Gender**				0.238
Male	17	12	10	
Female	18	23	25	
**Tooth**				0.179
Premolar	17	23	24	
Molar	18	12	11	
**Arch**				0.962
Maxillary	20	20	21	
Mandibular	15	15	14	

*: Significant at *P* ≤ 0.05

**Table 2:** Descriptive statistics and results of Kruskal-Wallis test for comparison of hypersensitivity (VAS) scores between the tested groups

Time	Group I (*n* = 35)		Group II (*n* = 35)		Group III (*n* = 35)		P value	Effect size (Eta squared)	
	Median (range)	Mean (SD)	Median (range)	Mean (SD)	Median (range)	Mean (SD)			
1 day	0 (0–3)	0.64 (1.15)	0 (0–3)	0.43 (1.09)	0 (0–2)	0.14 (0.53)	0.322	0.046	
1 week	0 (0–1)	0.14 (0.36)	0 (0–0)	0 (0)	0 (0–0)	0 (0)	0.129	0.1	
1 month	0 (0–0)	0 (0)	0 (0–0)	0 (0)	0 (0–0)	0 (0)	1		0

*: Significant at *P* ≤ 0.05

**Table 3:** Descriptive statistics and results of Friedman’s test for the changes in hypersensitivity (VAS) scores within each group

Time	Group I (*n* = 35)		Group II (*n* = 35)		Group III (*n* = 35)	
	Median (range)	Mean (SD)	Median (range)	Mean (SD)	Median (range)	Mean (SD)
1 day	0 (0–3)	0.64 (1.15) ^A^	0 (0–3)	0.43 (1.09)	0 (0–2)	0.14 (0.53)
1 week	0 (0–1)	0.14 (0.36) ^B^	0 (0–0)	0 (0)	0 (0–0)	0 (0)
1 month	0 (0–0)	0 (0) ^B^	0 (0–0)	0 (0)	0 (0–0)	0 (0)
*P* value	0.024*		0.135		0.368	
Effect size (*w*)	0.265		0.143		0.071	

*: Significant at *P* ≤ 0.05, Different superscripts in the same column indicate statistically significant change by time

## Discussion

Preheating of uncured RC has emerged as a recent innovation in RC application, aiming to enhance the handling characteristics during placement. Prior to light activation, pre-heating RC was suggested to enhance the physical and mechanical properties, which is considered a great advantage improving handling and marginal adaptation by decreasing the viscosity of highly filled packable RC, in addition to the degree of conversion from the monomeric to polymeric state [34–43]. These enhancements provided by the preheated RC may positively affect the sealing ability of RC and in turn decrease the postoperative hypersensitivity, however, the effect of exposing the same RC syringe to multiple preheating cycles is questionable.

The results of this study showed non-significant differences between the VAS scores for postoperative hypersensitivity in the three groups (Table 2). The null hypothesis was accepted as repeated preheating the RC syringe from room temperature to 68ºC had no significant effect on the post-operative hypersensitivity scores. Through reviewing literature, it was proven that there is no significant effect of the procedure of preheating the RC before application, in comparison to the RC used at room temperature on the properties of RC [44, 45], especially volumetric shrinkage of the material [23]; Although these studies were done in vitro, these findings conducted are directly related to the resultant restoration, that affects post-operative pain encountered by the patient. In a study conducted by one of the authors 2022 [46], to assess the post-gel shrinkage strain (PGSS) of matrix modifying bulk fill RC without preheating and the effect of repeated preheating once, twice and for three cycles at temperature of 68ºC compared to that tested at room temperature (control). It was found that repeated preheating cycles of matrix modifying bulk-fill RC prior to photo-activation had no adverse effect on the induced post-gel shrinkage strain. This Finding can support the use of repeated preheating technique without the fear of affection of the RC matrix, and in turn the adaptation quality of RC, which will influence the post-operative pain.

Although the highest recorded VAS score was 3 in all groups on the first day, however, the recorded scores are considered very low to be documented as severe pain that needed interpretation, even in group I which recorded statistical significance when compared to the other two follow up intervals. All the tested groups recorded regression of the VAS scores through the whole follow-up period which is in agreement with a clinical trial using a single preheating cycle [30] that recommended preheating of RC to increase the adaptability, handling properties of the material, and decrease gap formation, which are also highly related to the induced post-operative pain.

Comparing the results of groups II and III where group II subjected to one preheating cycle while group III subjected to ten cycles, there was almost zero VAS scores with non-significant difference between the two groups (Table 2), this supported the acceptance of the null hypothesis. Additionally, there were no significant differences between the three tested groups by the end of the follow-up period. These multiple zero score in the VAS test can also prove that the dentin bridge over the vital pulp in the moderate cavities can compensate for the increased temperature of the RC material used [47]. Additionally, the elevated temperature of RC affecting dentin and pulp was reported in literature to be below the critical temperature of the dentin and pulp complex, which is believed to be 5.5 °C above the normal pulp temperature [48]. In a clinical trial by one of the authors 2022 [49], evaluating the effect of preheating on two bulk fill RC; one Bis-GMA free and the other was Bis-GMA containing, at 50ºC and 70ºC, on the pulpal floor of prepared cavities and RC restoration temperatures. It was found that with pre-heating RC to 50ºC, the pulpal floor temperature did not reach even the normal body temperature. The preheated RC did not transmit heat to the pulpal floor above the critical level. Consequently, this technique is extremely safe to be used. However, only a slight increase in the temperature of the RC from 6 to 8ºC was found when applied clinically in the prepared cavities in vivo even when the heating temperature reached 68ºC [50], which supports the absence of negative effect of preheating the RC on dentin-pulp complex and in consequence the post operative pain.

Applying Bulk-fill RC for class II posterior restorations was encouraged by many dentists to facilitate the restorative procedure and was proven to give superior clinical performance results comparable to conventional RC [17, 18]. Unfortunately, both the handling properties of most bulk-fill RCs, in addition to their adaptability to cavity walls, are not favourable especially while restoring compound cavities, due to the RC’s high viscosity [51–53]. In the current study, it was suggested to overcome the poor handling properties of the bulk-fill RC using the incremental packing technique rather than the single increment technique in addition to the preheating protocol utilized. The use of bulk-fill RC in an incremental packing technique was proved to enhance the degree of conversion, decrease the shrinkage stress and also for better transmission of the curing light in the deeper portions of the cavities with better marginal adaptation [54], which may provoke the use of bulk-fill RCs even with no preheating process but in an incremental build-up protocol [55, 56].

Clinically, there is great drop in the temperature of the RC syringe once it is removed from the heating apparatus until it is inserted in the prepared cavity if this procedure takes more than 30 s [27, 57]. To avoid temperature drop, especially with the incremental technique, only two increments for cavity restoration were utilized, where packing consume 10 s and light curing consumes another 10 s for each increment. This may justify the low VAS scores reported by patients even in group I, where superior adaptation was achieved through incremental packing. On the other hand, it was found that the use of bulk-fill technique or incremental packing techniques shows no clinical significant difference in other literature [17, 52, 58].

Despite its improved properties that were proven in these in vitro studies, RC preheating has not been widely adopted as a regular clinical protocol. One possible explanation for dentists’ hesitation to use warmed composite is a lack of clinical data on the effect of this procedure specially on postoperative hypersensitivity. However, up to our knowledge, only three clinical trials were conducted to support the RC preheating, but only once before application [27, 30, 53]. However, there is lack of data regarding the effect of repeated preheating on the postoperative hypersensitivity encountered by patients. Thus, a critique of the results of this study in terms of the present literature cannot be accomplished.

Although an in vitro study proved that the properties of the RC material are not affected by multiple cycles of preheating up to 40 cycles [59], ten cycles of RC preheating were chosen in the current study to represent the number of compound cavities that the average RC syringe can offer. Fortunately, the RC used was Filtek One Bulk which is recommended by the manufacturer to be preheated before use to have this technique’s advantages.

Studying the effect of many preheating cycles may confirm the idea and convince the dentists to preheat RCs before use without fear. While it is still not easy to convince dentists to preheat RCs before use, the problem becomes more complicated if they were asked to use the same RC syringe for several patients with multiple exposures to the raised temperature, believing that this could affect the quality of the material and its physical and mechanical properties. Dentists may be less hesitant if a RC compule is used for a single patient with a single increase in temperature before use, as recommended by some manufacturers (VisCalor Bulk, Voco), which in an economic point of view is going to be of higher cost when compared to RC syringes. However, an in vitro study proved that the properties of the RC material is not affected by multiple cycles of preheating up to 40 cycles [59].

One of the major limitations of this clinical trial is that one month could be a short period for observing substantial changes. Thus, a long-term clinical evaluation may be able to better assess the effect of preheating bulk-fill RCs, taking in concern the marginal adaptation and integrity which may be affected by time and consequently affect the pain felt by the patients in the form of a complaint of delayed hypersensitivity.

## Conclusion

Under the limitations of this study, it could be concluded that after one month, the preheated bulk fill RC for single and ten cycles had no adverse effect on post-operative hypersensitivity. Although the three tested groups were recording higher VAS scores on the first day, none of the three tested groups had VAS scores more than 3, which is highly accepted clinically, with no required interpretation.

### Clinical relevance

Nowadays, with the great spread of RC use in different dental restorations, by continually preheating the RC syringe, the post-operative hypersensitivity encountered by patients is not affected. This knowledge could be extremely important since the same RC syringe can be preheated multiple times before it is used up, with the benefit of a temporary drop in viscosity that enhances adaptability to the cavity, preventing or even reducing post-operative hypersensitivity, and having the advantage of the superior handling properties of the preheated RC.

## Data Availability

The datasets used and/or analysed during the current study are available from the corresponding author on reasonable request.
